# Metabolomic biomarkers are associated with mortality in patients with cirrhosis caused by primary biliary cholangitis or primary sclerosing cholangitis

**DOI:** 10.2144/fsoa-2019-0124

**Published:** 2019-12-17

**Authors:** Ayse L Mindikoglu, Cristian Coarfa, Antone R Opekun, Vijay H Shah, Juan P Arab, Konstantinos N Lazaridis, Nagireddy Putluri, Chandrashekar R Ambati, Matthew J Robertson, Sridevi Devaraj, Prasun K Jalal, Abbas Rana, John A Goss, Thomas C Dowling, Matthew R Weir, Stephen L Seliger, Jean-Pierre Raufman, David W Bernard, John M Vierling

**Affiliations:** 1 Margaret M & Albert B Alkek Department of Medicine, Section of Gastroenterology & Hepatology, Baylor College of Medicine, Houston, TX, USA; 2 Michael E DeBakey Department of Surgery, Division of Abdominal Transplantation, Baylor College of Medicine, Houston, TX, USA; 3 Department of Molecular & Cellular Biology, Baylor College of Medicine, Houston, TX, USA; 4 Dan L Duncan Cancer Center, Baylor College of Medicine, Houston, TX, USA; 5 Department of Pediatrics, Division of Gastroenterology, Nutrition & Hepatology, Baylor College of Medicine, Houston, TX, USA; 6 Department of Medicine, Division of Gastroenterology & Hepatology, Mayo Clinic College of Medicine, Rochester, MN, USA; 7 Departamento de Gastroenterologia, Escuela de Medicina, Pontificia Universidad Catolica de Chile, Santiago, Chile; 8 Clinical Chemistry & Point of Care Technology, Texas Children's Hospital & Health Centers, Department of Pathology & Immunology, Baylor College of Medicine, Houston, TX, USA; 9 Ferris State University, College of Pharmacy, Grand Rapids, MI, USA; 10 Department of Medicine, Division of Nephrology, University of Maryland School of Medicine, Baltimore, MD, USA; 11 Department of Medicine, Division of Gastroenterology & Hepatology, University of Maryland School of Medicine, Baltimore, MD, USA; 12 Department of Pathology & Genomic Medicine, Houston Methodist Hospital, Houston, TX, USA

**Keywords:** biomarker, cirrhosis, liver transplantation, MELD-Na score, metabolite, metabolomics, mortality, myo-inositol, primary biliary cholangitis, primary sclerosing cholangitis

## Abstract

**Aim:**

To assess the ability of signature metabolites alone, or in combination with the model for end-stage liver disease-Na (MELD-Na) score to predict mortality in patients with cirrhosis caused by primary biliary cholangitis or primary sclerosing cholangitis.

**Materials & methods:**

Plasma metabolites were detected using ultrahigh-performance liquid chromatography/tandem mass spectrometry in 39 patients with cirrhosis caused by primary biliary cholangitis or primary sclerosing cholangitis. Mortality was predicted using Cox proportional hazards regression and time-dependent receiver operating characteristic curve analyses.

**Results:**

The top five metabolites with significantly greater accuracy than the MELD-Na score (area under the receiver operating characteristic curve [AUROC] = 0.7591) to predict 1-year mortality were myo-inositol (AUROC = 0.9537), N-acetylputrescine (AUROC = 0.9018), trans-aconitate (AUROC = 0.8880), erythronate (AUROC = 0.8345) and N6-carbamoylthreonyladenosine (AUROC = 0.8055). Several combined MELD-Na-metabolite models increased the accuracy of predicted 1-year mortality substantially (AUROC increased from 0.7591 up to 0.9392).

**Conclusion:**

Plasma metabolites have the potential to enhance the accuracy of mortality predictions, minimize underestimates of mortality in patients with cirrhosis and low MELD-Na scores, and promote equitable allocation of donor livers.

## Lay abstract

To receive a liver transplant, patients with cirrhosis need to be listed on the US liver transplant waiting list based on a score called the model for end-stage liver disease-Na (MELD-Na) score that is expected to accurately rank the patients based on urgency for a liver transplant. However, MELD-Na score is not sufficiently accurate to identify many patients with cirrhosis with the highest urgency, and this results in longer waiting times on the liver transplant list, and therefore higher death rates. We identified several metabolomic biomarkers that can increase the accuracy of the MELD-Na score, and optimize the allocation of donor livers for transplantation of patients with cirrhosis.

In the current US liver transplant system, donor livers are allocated to patients with cirrhosis based on their model for end-stage liver disease-Na (MELD-Na) scores [1], which estimates the mortality within 90 days. Despite the expectation that the MELD-Na score accurately predicts mortality within 90 days and facilitates allocation to patients with a higher risk of death on the liver transplant waiting list, recent analyses indicate otherwise. Analysis of organ procurement and transplantation network data showed that the accuracy of MELD-Na score has declined from the area under the receiver operating characteristic curve (AUROC) of 0.78 in 2004 to 0.70 in 2015 [2]. This drop in applicability of MELD-Na score as a predictor of 90-day mortality reflected the concurrent decline in liver transplant listings for cirrhosis caused by chronic hepatitis C. Failure to accurately predict 90-day mortality in 30% of patients with cirrhosis on the liver transplant waiting list using the MELD-Na score extends their waiting times and increases the risk of death while waiting for liver transplantation. In addition, the inaccuracy of the MELD-Na score may result in failure to refer patients with cirrhosis to liver transplant centers, whose true risk of dying requires consideration of liver transplantation.

To address the urgent need for biomarkers that more accurately predict mortality in patients with cirrhosis, we previously reported that 34 plasma metabolites were significant predictors of hepatorenal dysfunction and mortality in adult patients with cirrhosis caused primarily by chronic hepatitis C, alcohol and nonalcoholic steatohepatitis [3]. Since this patient cohort did not include sufficient numbers of patients with cirrhosis caused by chronic cholestatic liver diseases, the goal of the present study was to assess whether our previously identified plasma metabolites also predicted mortality in patients with cirrhosis caused by the two most common adult cholestatic liver diseases, primary biliary cholangitis (PBC) and primary sclerosing cholangitis (PSC). Thus, the goal of the present study was to assess the performance of our previously identified plasma metabolic signature in adult patients with cirrhosis caused by PBC or PSC in order to answer three key questions. First, does the plasma metabolomic signature identified in patients with cirrhosis caused by noncholestatic disease etiologies accurately predict hepatorenal dysfunction in patients with cirrhosis caused by two cholestatic liver diseases, including PBC and PSC? Second, is there an optimal metabolomic signature that more accurately predicts mortality than the MELD-Na score in cirrhosis caused by PBC or PSC? At last, does a combination of a metabolite with the MELD-Na score in an additive mathematical prognostic model predict mortality more accurately than the MELD-Na score alone in patients with cirrhosis caused by PBC or PSC?

## Materials & methods

Plasma samples from 39 patients with PBC (n = 13) and PSC (n = 26) cirrhosis were provided by the Mayo Clinic College of Medicine. The Institutional Review Boards of the Baylor College of Medicine and Mayo Clinic College of Medicine approved to plan for analysis of plasma samples and patient-related data.

### Methods for detection, identification & measurement of plasma metabolites

Freshly-thawed plasma samples were analyzed at Baylor College of Medicine Metabolomics Core facility. Metabolites were extracted from plasma using previously described standard procedures for targeted metabolomic profiling using ultrahigh-performance liquid chromatography (UPLC)-tandem mass spectrometry [4–8]. Pooled plasma samples were used as quality controls. For extraction of the metabolome, 100 μl of plasma was mixed with a methanol mixture containing equimolar amounts of eight internal standard compounds, and metabolic extraction was performed using consecutive application of ice-cold organic and aqueous solvents (water: methanol: chloroform: water, with a ratio of 1:4:3:1) followed by deproteinization and drying of the extract. The dried extract was resuspended in injection solvent and analyzed using UPLC-tandem mass spectrometry (Agilent 1290 series UPLC system equipped with a degasser, binary pump, thermostatted autosampler and column oven, Agilent Technologies, CA, USA). The multiple reaction monitoring-based measurement of relative metabolite levels was performed using normal phase chromatographic separation. All samples were kept at 4°C, and analysis was performed on aliquots of 5 and 10 μl.

#### Separation of metabolites

Two methods were used to separate metabolites. In Method 1, mass spectrometry employed electron spray ionization in the positive mode. Metabolites were separated using a Waters XBridge Amide 3.5 μm, 4.6 × 100 mm UPLC column (Waters, MA, USA). For the chromatographic separation, mobile phase A used 0.1% formic acid in water, and mobile phase B used acetonitrile. Chromatographic separation was performed using the following gradient: 0–3 min 85% B; 3–12 min 30% B, 12–15 min 2% B, 16 min 95% B, followed by re-equilibration until the end of the gradient 23 min to the initial starting condition of 85% B. The flow rate of the solvents was set at 0.3 ml/min, and the injection volume was 5 μl.

In Method 2, mass spectrometry employed negative mode electron spray ionization. Metabolites were separated using Waters XBridge Amide 3.5 μm, 4.6 × 100 mm UPLC column (Waters). For chromatographic separation, mobile phase A used 20 mM ammonium acetate in water with pH 9.0 and mobile phase B used 100% acetonitrile. Chromatographic separation was performed using the following gradient: 0–3 min 85% B, 3–12 min 30% B, 12–15 min 2% B, 15–16 min 85% B followed by re-equilibration until the end of the gradient 23 min to the initial starting condition of 85% B. The flow rate of the solvents was set at 0.3 ml/min, and an injection volume was 10 μl.

#### Metabolomic data

The metabolomics pipeline was monitored for its reproducibility and robustness using two sets of controls. These included a spiked internal standard (15N-labeled tryptophan) and a matrix pool (mouse liver pool). At the beginning of the metabolic extraction process, known amounts of this standard were spiked into the test samples and the matrix pool. Three samples of the matrix pool were co-extracted along with the test samples following identical extraction procedures. During the mass spectrometry analysis phase, the three matrix pools were examined at the beginning, middle and end of the sample run. Data analysis examined the coefficient of variation for spiked tryptophan in both the matrix pool and test samples. The coefficient of variation was found to be approximately 6%. Furthermore, the peak area for the candidate metabolite in each sample was normalized to the peak area for the spiked tryptophan standard, followed by log_2_ transformation of the resulting ratio.

### Sample size

With 39 samples, assuming a standard deviation of 50% of the population mean, a fold change of 2 can be detected at significance level α = 0.05 with a power of 99.86%, and a fold change of 1.5 signifies a significance level α = 0.05 with a power of 96.56%.

### Data analysis

All statistical analyses were performed using SAS Version 9.4 TS Level 1M5 X64_10PRO platform (SAS, NC, USA) [9] and R software [10]. We used log_2_ values of the plasma metabolites. A two-tailed p < 0.05 was considered statistically significant.

#### Specific research questions

Can plasma metabolites accurately predict hepatorenal dysfunction in patients with cirrhosis caused by PBC or PSC? We previously reported a significant association between specific plasma metabolites and hepatorenal dysfunction in adults with cirrhosis caused primarily by noncholestatic liver diseases [3]. The present study investigated the potential of these individual plasma metabolites, alone or in combination with the MELD-Na score, as biomarkers predictive of hepatorenal dysfunction in 39 adult patients with either PBC or PSC cirrhosis. Because an assay for C-glycosyltryptophan was no longer commercially available, we tested only 33 of the 34 plasma metabolites in the previously reported metabolomic signature.

Patients were stratified based on clinical and laboratory categories that defined low and high severity of liver and kidney disease, described previously [3]. Categories included ascites status (absent vs present), MELD-Na score groups (i.e., scores 6–9, 10–19, 20–40), estimated glomerular filtration rate (eGFR) groups (eGFR <60 vs ≥60 ml/min/1.73 m^2^, eGFR was calculated using the four-variable modification of diet in renal disease study equation [11]), and above versus below the median values of GFR biomarkers (serum Cr and cystatin C) and MELD-Na score (calculated using serum total bilirubin, Cr, Na and international normalized ratio). We defined the fold-change as the change in the mean value of a plasma metabolite from low-disease severity to the high-disease severity category. Furthermore, we identified the highest fold-change based on the mean fold-change of all mean fold changes across comparisons for the six clinical and laboratory categorical variables. We used Student’s t-test to determine statistically significant fold changes in metabolite values between low and high liver and kidney disease severity groups and used Benjamini–Hochberg method [12], as implemented in the R statistical system, to adjust p-values for false discovery rate [10]. Fold changes were considered to be statistically significant if the Q value was <0.25. For variables with more than two categories, such as MELD-Na score classes, we reported a metabolite based on prespecified criteria: evidence of statistical significance across comparisons between any pair of patient groups; a consistent direction of change between low versus high disease severity when statistically significant across multiple comparisons; and the selection of categorical clinical variables based on the highest fold-change in the mean value of the metabolite between any pair of groups. Within each independent clinical and laboratory variable category, we generated comparisons for each metabolite.

Can plasma metabolites predict mortality more accurately than the MELD-Na score in patients with cirrhosis caused by PBC or PSC? Can a combination of a metabolite with the MELD-Na score in an additive mathematical prognostic model predict mortality more accurately than the MELD-Na score alone in patients with cirrhosis caused by PBC or PSC? Follow-up time was defined as the time interval between the date of blood collection and date of death, liver transplantation or the last clinical encounter. Individuals were censored at the time of the last clinical encounter or at the time of liver transplantation. Hazard ratios were calculated using Cox proportional hazards regression models [13]. To assess the performance of Cox models using plasma metabolites alone or in combination with and MELD-Na score to predict 1-year mortality, we computed the time-dependent AUROC [14] and 95% confidence limits of AUROC by inverse probability using a censoring weighting method [9]. We used a time-dependent AUROC analysis instead of a standard AUROC analysis [15] to minimize problems of standard AUROC analyses in which survival outcomes and metabolite levels are considered to be fixed over the study period [15]. CIs for AUROC that did not include 0.50 were considered significant.

## Results

### Study population

Patient characteristics are shown in Table 1. Among the 39 patients with cirrhosis resulting from PBC (n = 13; 33%) or PSC (n = 26; 67%), 56% were women and 100% were Caucasian. Over 60% of patients had ascites, and over 50% of patients had a MELD-Na score ranging between 10 and 19. During the study period, 26% of patients were alive, 28% were dead and 46% were transplanted. The mean follow-up time (interval between the date of blood collection and date of death, liver transplantation or the last clinical encounter) was 3.09 years (standard deviation [SD] = 2.94 years). The shortest follow-up time was 0.07 year and the longest follow-up time was 12.2 years.

**Table 1.** Characteristics of 39 patients with cirrhosis caused by primary biliary cholangitis or primary sclerosing cholangitis.

Characteristics		n = 39 (%)
Diagnosis	– PBC	13 (33)
	– PSC	26 (67)
Gender	– Male	17 (44)
	– Female	22 (56)
Race	Caucasian	39 (100)
Ascites	Present	24 (62)
MELD-Na score	– 6–9	11 (28)
	– 10–19	20 (51)
	– 20–40	8 (21)
eGFR (ml/min/1.73 m^2^)^†^	– Stage 1 (≥90)	12 (31)
	– Stage 2 (≥60 and <90)	14 (36)
	– Stage 3 (≥30 and <60)	12 (31)
	– Stage 4 (≥15 and <30)	1 (3)
Survival outcomes	– Alive	10 (26)
	– Death	11 (28)
	– Liver transplant	18 (46)
Hepatic malignancy	– Hepatocellular carcinoma	4 (10)
	– Cholangiocarcinoma	3 (8)
	– Both	3 (8)
	– None	29 (74)
		Mean (SD)
Age (y)		57.64 (11.85)
MELD-Na score		13.97 (6.82)
Total bilirubin (mg/dl)		3.46 (6.16)
International normalized ratio		1.36 (0.67)
Serum Na (mmol/l)		137.72 (3.94)
Serum Cr (mg/dl)		1.01 (0.39)
Serum cystatin C (mg/l)		1.13 (0.46)
Plasma symmetric dimethylarginine (micromole/l)		0.52 (0.31)

† Estimated using MDRD-4 equation [11].

eGFR: Estimated glomerular filtration rate; MDRD: Modification of diet in renal disease; MELD-Na: Model for end-stage liver disease-Na; PBC: Primary biliary cholangitis; PSC: Primary sclerosing cholangitis; SD: Standard deviation.

### Associations between metabolites & hepatorenal dysfunction

**Figure 1.**
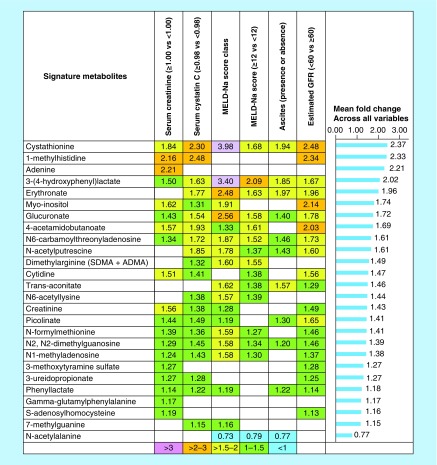
Twenty-five of 33 metabolites were significantly increased and associated with at least one of the six clinical and laboratory variable categories indicative of liver and kidney disease severity in 39 patients with cirrhosis caused by primary biliary cholangitis or primary sclerosing cholangitis. The highest mean fold-change (2.37) occurred with cystathionine when patients with low liver and kidney disease severity were compared with those with high disease severity across six clinical variables. The lowest significant positive mean fold-change (1.15) occurred with 7-methylguanine. Only one metabolite, N-acetylalanine, was significantly associated with disease severity but with a decreased mean fold-change (0.77). The corresponding cell was left blank if a metabolite did not show a significant association with the clinical and laboratory variable. GFR: Glomerular filtration rate; MELD-Na: Model for end-stage liver disease-Na.

Plasma levels of 25 of 33 previously identified metabolites were significantly increased in patients with high liver and kidney disease severity compared with those with low liver and kidney disease. Elevated plasma levels of metabolites were associated with at least one of the six clinical and laboratory variable categories indicative of the severity of kidney or liver dysfunction (Figure 1).

### Comparison of MELD-Na score & single metabolite models as predictors of mortality

**Figure 2.**
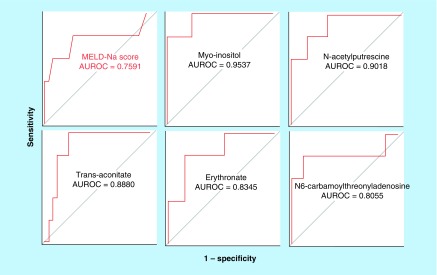
The area under the receiver operating characteristics curves of the top five single metabolite models, including myo-inositol, N-acetylputrescine, trans-aconitate, erythronate and N6-carbamoylthreonyladenosine versus area under the receiver operating characteristics curves of the model for end-stage liver disease-Na score predicting 1-year mortality in patients with cirrhosis caused by primary biliary cholangitis or primary sclerosing cholangitis. Several plasma metabolites had significantly higher accuracy to predict 1-year mortality compared with the accuracy of the MELD-Na score. AUROC: Area under the receiver operating characteristic.

The accuracy of the MELD-Na score as a predictor of 1-year mortality was only fair (AUROC = 0.7591, 95% CI: 0.5553–0.9628). In contrast, plasma levels of several metabolites had a greater accuracy to predict 1-year mortality than the MELD-Na score (Table 2 & Figure 2). The top five plasma metabolites with a greater predictive accuracy than the MELD-Na score were myo-inositol (AUROC = 0.9537, 95% CI: 0.5612–1.000), N-acetylputrescine (AUROC = 0.9018, 95% CI: 0.7763–1.000), trans-aconitate (AUROC = 0.8880, 95% CI: 0.7149–1.000), erythronate (AUROC = 0.8345, 95% CI: 0.7094–0.9595) and N6-carbamoylthreonyladenosine (AUROC = 0.8055, 95% CI: 0.5991–1.000).

**Table 2.** Performance of single metabolite models versus model for end-stage liver disease-Na score predicting 1-year mortality in 39 patients with cirrhosis caused by primary biliary cholangitis or primary sclerosing cholangitis.

Plasma metabolites	Hazard ratio^†^	p-value for hazard ratio	Time-dependent AUROC (at 1 year)^‡^	AUROC 95% confidence limits (lower)^§^	AUROC 95% confidence limits (upper)^§^
Myo-inositol	4.967	0.0013	0.9537	0.5612	1.0000
N-Acetylputrescine	3.343	0.0052	0.9018	0.7763	1.0000
Trans-aconitate	2.080	0.0181	0.8880	0.7149	1.0000
N-Acetylalanine	0.040	0.0009	0.8438	0.1627	1.0000
Erythronate	7.595	0.0010	0.8345	0.7094	0.9595
N1-Methyladenosine	13.113	0.0038	0.8279	0.3914	1.0000
N6-Carbamoylthreonyladenosine	13.420	0.0002	0.8055	0.5991	1.0000
N2,N2-Dimethylguanosine	27.828	0.0003	0.8055	0.5816	1.0000
Pseudouridine	0.732	0.6581	0.7837	0.2180	1.0000
Glucuronate	4.590	0.0004	0.7828	0.5997	0.9659
Dimethylarginine (SDMA + ADMA)	5.255	0.0087	0.7790	0.5566	1.0000
N4-Acetylcytidine	0.759	0.1244	0.7716	0.5108	1.0000
MELD-Na score	**1.162**	**0.0020**	**0.7591**	**0.5553**	**0.9628**
Picolinate	2.628	0.1060	0.7510	0.5272	0.9748
Creatinine	3.741	0.0605	0.7449	0.1407	1.0000
3-(4-Hydroxyphenyl)lactate	2.951	0.0056	0.7288	0.4799	0.9777
3-Methoxtyrosine	0.139	0.0066	0.7248	0.3760	1.0000
Xylitol	4.130	0.1032	0.7151	0.1167	1.0000
Cystathionine	1.972	0.0143	0.7150	0.4541	0.9759
1-Methylhistidine	1.967	0.0097	0.6893	0.4248	0.9537
Cytidine	2.516	0.0690	0.6867	0.2664	1.0000
4-Acetamidobutanoate	2.547	0.0007	0.6649	0.4267	0.9032
Gamma-glutamylphenylalanine	0.569	0.5629	0.6626	0.1644	1.0000
N-Formylmethionine	3.838	0.0851	0.6591	0.2915	1.0000
N-Acetylserine	1.185	0.5744	0.6462	0.3149	0.9775
S-Adenosylhomocysteine	2.132	0.4237	0.6205	0.0874	1.0000
Adenosine	0.800	0.1537	0.5857	0.2650	0.9064
Phenyllactate	5.234	0.0998	0.5775	0.0838	1.0000
Adenine	1.252	0.3103	0.5713	0.1576	0.9849
N6-Acetyllysine	1.271	0.6092	0.5489	0.3094	0.7884
7-Methylguanine	2.535	0.3854	0.5290	0.0823	0.9757
3-Methoxytyramine Sulfate	1.425	0.5396	0.5109	0.1052	0.9167
N-Acetylvaline	0.803	0.4900	0.4993	0.1065	0.8922
3-Ureidopropionate	0.867	0.8197	0.4098	0.0000	0.8829

Bold font highlights the MELD-Na score.

† Metabolites were expressed on a log_2_ scale – hazard ratios represent the risk of death associated with one log_2_ unit increment.

‡ Data were sorted by AUROC (largest to smallest), then by p-value (smallest to largest).

§ CIs that did not include 0.50 were considered significant (e.g. 95% CIs for the AUROC of myo-inositol model is significant because it did not include 0.50). Several plasma metabolites predicted 1-year mortality with significantly greater accuracy (AUROC) compared with the accuracy (AUROC) of the MELD-Na score.

¶ SDMA; ADMA.

ADMA: Asymmetric dimethylarginine; AUROC: Area under the receiver operating characteristic curve; MELD-Na: Model for end-stage liver disease-Na; SDMA: Symmetric dimethylarginine.

### Performance of models combining the MELD-Na score & specific plasma metabolite as predictors of mortality

**Figure 3.**
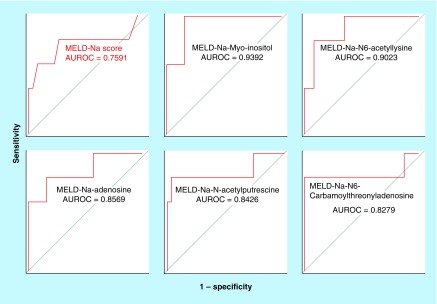
The area under the receiver operating characteristics curves of the top five model for end-stage liver disease-Na-metabolite models, including model for end-stage liver disease-Na-myo-inositol, model for end-stage liver disease-Na-N6-acetyllysine, model for end-stage liver disease-Na-adenosine, model for end-stage liver disease-Na-N-acetylputrescine, and model for end-stage liver disease-Na-N6-carbamoylthreonyladenosine versus area under the receiver operating characteristics curve of the model for end-stage liver disease-Na score alone predicting 1-year mortality in patients with cirrhosis caused by primary biliary cholangitis or primary sclerosing cholangitis. Several models that combined the MELD-Na score with a plasma metabolite had significantly higher accuracy to predict 1-year mortality compared with the accuracy of the MELD-Na score alone. AUROC: Area under the receiver operating characteristic; MELD-Na: Model for end-stage liver disease-Na.

Several bivariate combinations of MELD-Na score and plasma metabolites had a greater accuracy to predict 1-year mortality than the MELD-Na score alone (Table 3 & Figure 3). The top five performing MELD-Na-metabolite models were MELD-Na-myo-inositol (AUROC = 0.9392; 95% CI: 0.5804–1.000), MELD-Na-N6-acetyllysine (AUROC = 0.9023; 95% CI: 0.7442–1.000), MELD-Na-adenosine (AUROC = 0.8569; 95% CI: 0.5918–1.000), N-acetylputrescine (AUROC = 0.8426; 95% CI: 0.6578–1.000) and N6-carbamoylthreonyladenosine (AUROC = 0.8279; 95% CI: 0.6246–1.000). The predictive accuracy of each of these five combination MELD-Na-metabolite models exceeded that of the predictive accuracy of the MELD-Na score alone (AUROC = 0.7591; 95% CI: 0.5553–0.9628).

**Table 3.** Performance of model for end-stage liver disease-Na-metabolite models versus model for end-stage liver disease-Na score to predict 1-year mortality in patients with cirrhosis caused by primary biliary cholangitis or primary sclerosing cholangitis.

MELD-Na Score		Plasma metabolites	Hazard ratio for MELD-Na score^†^	p-value for hazard ratio for MELD-Na score	Hazard ratio for metabolite^†^	p-value for hazard ratio for metabolite	Time-dependent AUROC (at year)^‡^	AUROCC 95% confidence limits (lower)^§^	AUROCC 95% confidence limits (upper)^§^
MELD-Na score	+	Myo-inositol	1.091	0.1104	3.139	0.0356	0.9392	0.5804	1.0000
MELD-Na score	+	N6-Acetyllysine	1.204	0.0026	0.508	0.2777	0.9023	0.7442	1.0000
MELD-Na score	+	N-Acetylalanine	1.052	0.4350	0.078	0.0423	0.8653	0.1290	1.0000
MELD-Na score	+	Gamma-Glutamylphenylanine	1.163	0.0021	0.530	0.5244	0.8574	0.1634	1.0000
MELD-Na score	+	Adenosine	1.181	0.0018	0.722	0.0948	0.8569	0.5918	1.0000
MELD-Na score	+	N-Acetylputrescine	1.135	0.0162	2.422	0.0461	0.8426	0.6578	1.0000
MELD-Na score	+	N6-Carbamoylthreonyladenosine	1.091	0.1253	8.962	0.0050	0.8279	0.6246	1.0000
MELD-Na score	+	Erythronate	1.083	0.1803	4.269	0.0468	0.8193	0.6705	0.9681
MELD-Na score	+	3-Ureidopropionate	1.190	0.0008	0.312	0.2005	0.8190	0.3470	1.0000
MELD-Na score	+	N2,N2-Dimethylguanosine	1.105	0.0915	14.670	0.0064	0.8055	0.5803	1.0000
MELD-Na score	+	N1-Methyladenosine	1.135	0.0246	5.320	0.0632	0.7975	0.3781	1.0000
MELD-Na score	+	N-Acetylvaline	1.172	0.0020	0.724	0.3344	0.7900	0.5956	0.9844
MELD-Na score	+	S-Adenosylhomocysteine (SAH)	1.185	0.0017	3.375	0.1941	0.7889	0.3442	1.0000
MELD-Na score	+	Glucuronate	1.081	0.1663	3.402	0.0138	0.7826	0.5927	0.9724
MELD-Na score	+	Dimethylarginine (SDMA + ADMA)^¶^	1.124	0.0355	3.257	0.0665	0.7802	0.5775	0.9829
MELD-Na score	+	Creatinine	1.209	0.0011	5.045	0.0157	0.7748	0.2986	1.0000
MELD-Na score	+	Trans-aconitate	1.149	0.0267	1.112	0.7862	0.7741	0.6183	0.9300
MELD-Na score	+	Picolinate	1.154	0.0035	2.130	0.2150	0.7741	0.5740	0.9743
MELD-Na score	+	Cystathionine	1.124	0.0398	1.418	0.3167	0.7674	0.5597	0.9750
MELD-Na score	+	Adenine	1.177	0.0012	1.425	0.1446	0.7671	0.5667	0.9676
MELD-Na score	+	Xylitol	1.138	0.0097	2.081	0.3330	0.7671	0.3421	1.0000
MELD-Na score	+	N-Acetylserine	1.169	0.0023	1.219	0.5192	0.7662	0.5989	0.9335
MELD-Na score	+	Cytidine	1.152	0.0049	1.872	0.1996	0.7594	0.5354	0.9834
MELD-Na score	+	Pseudouridine	1.164	0.0018	0.597	0.4666	0.7594	0.5467	0.9721
MELD-Na score			**1.162**	**0.0020**			**0.7591**	**0.5553**	**0.9628**
MELD-Na score	+	N4-Acetylcytidine	1.168	0.0074	1.040	0.8623	0.7442	0.5877	0.9007
MELD-Na score	+	N-Formylmethionine	1.146	0.0075	2.062	0.4006	0.7440	0.5339	0.9541
MELD-Na score	+	3-(4-Hydroxyphenyl)lactate	1.098	0.1204	1.932	0.1702	0.7440	0.5479	0.9400
MELD-Na score	+	7-Methylguanine	1.168	0.0020	2.651	0.3461	0.7365	0.4934	0.9796
MELD-Na score	+	3-Methoxytyramine Sulfate	1.159	0.0026	1.255	0.7981	0.7365	0.3450	1.0000
MELD-Na score	+	1-Methylhistidine	1.137	0.0107	1.758	0.0530	0.7358	0.5320	0.9395
MELD-Na Score	+	Phenyllactate	1.146	0.0071	2.881	0.3779	0.7213	0.0924	1.0000
MELD-Na score	+	4-Acetamidobutanoate	1.142	0.0216	1.934	0.0168	0.7126	0.5147	0.9106
MELD-Na score	+	3-Methoxtyrosine	1.105	0.0707	0.319	0.1265	0.6883	0.4361	0.9405

Bold font highlights the MELD-Na score.

† Metabolites were expressed on a log2 scale - hazard ratios represent the risk of death associated with one log_2_ unit increment.

‡ Data were sorted by AUROC (largest to smallest), then by p-value (smallest to largest).

§ CIs that did not include 0.50 were considered significant (e.g., 95% CIs for the AUROC of MELD-Na-myo-inositol model was significant because it did not include 0.50). Several models that combined MELD-Na score with a plasma metabolite predicted 1-year mortality with significantly greater accuracy (AUROC) compared with the accuracy (AUROC) of the MELD-Na score alone.

¶ SDMA; ADMA.

ADMA: Asymmetric dimethylarginine; AUROC: Area under the receiver operating characteristic; MELD-Na: Model for end-stage liver disease-Na; SDMA: Symmetric dimethylarginine.

Even after controlling for MELD-Na score, several plasma metabolites were significantly associated with mortality. These metabolites were myo-inositol, N-acetylalanine, N-acetylputrescine, N6-carbamoylthreonyladenosine, erythronate, N2,N2-dimethylguanosine, glucuronate, creatinine and 4-acetamidobutanoate (Table 3).

To determine the impact of hepatobiliary malignancy on these results, we performed a sensitivity analysis on 29 patients with PBC or PSC without hepatobiliary malignancies. Among these 29 patients, the predictive accuracy of the MELD-Na score for 1-year mortality was poor: hazard ratio (HR) = 1.127; p = 0.0804; AUROC = 0.6439; 95% CI: 0.3427–0.9452. The top three metabolites retaining the highest accuracy were myo-inositol (HR = 4.181; p = 0.0163; AUROC = 0.9394; 95% CI: 0.4760–1.000), trans-aconitate (HR = 12.922; p = 0.0127; AUROC = 0.9242; 95% CI: 0.6237–1.000) and erythronate (HR = 5.317, p = 0.0242; AUROC = 0.7879; 95% CI: 0.5003–1.000). The top five MELD-Na-metabolite models identified for patients without malignancies were: MELD-Na-myo-inositol (AUROC = 0.9545; 95% CI: 0.5483–1.000), MELD-Na-N4-acetylcytidine (AUROC = 0.9545; 95% CI: 0.7192–1.000), MELD-Na-N6-acetyllysine (AUROC = 0.9394; 95% CI: 0.6718–1.000), trans-aconitate (AUROC = 0.9091; 95% CI: 0.5280–1.000) and N-acetylserine (AUROC = 0.8182; 95% CI: 0.5823–1.000).

## Discussion & conclusion

The principal finding of this study demonstrates that plasma metabolites are accurate, predictive biomarkers of hepatorenal dysfunction and mortality in adults with cirrhosis caused by the two most common cholestatic liver diseases, PBC and PSC. These results extend our prior observations of the utility of plasma metabolite biomarkers in adult patients with cirrhosis caused by noncholestatic liver diseases [3]. Specifically, the fold changes of 26 of 33 measured plasma metabolites were statistically significant when when patients with PBC or PSC cirrhosis who had high liver and kidney disease severity were compared with those with low liver and kidney disease severity (Figure 1).

In addition, several plasma metabolites were more accurate predictors of 1-year mortality than the MELD-Na score in patients with cirrhosis caused by PBC or PSC. The top five plasma metabolites included myo-inositol, N-acetylputrescine, trans-aconitate, erythronate and N6-carbamoylthreonyladenosine (Table 2 & Figure 2). Of note, several plasma metabolites were superior predictors of 1-year mortality than plasma Cr, which is a principal component of the MELD-Na score (Table 2).

The metabolite myo-inositol was the most accurate biomarker to predict 1-year mortality, exceeding the predictive accuracy of all other plasma metabolites, as well as the MELD-Na score. Myo-inositol, a carbocyclic sugar that is an essential constituent of phosphatidylinositol in cell membranes, mediates cell signal transduction in response to several hormones, neurotransmitters and growth factors and regulates intracellular osmolality [16–22]. Thus, its elevation in patients with PBC or PSC cirrhosis may reflect dysfunctional membrane integrity, intracellular signaling and/or osmoregulation. Increased plasma myo-inositol is also a biomarker of renal dysfunction [3,17,23,24]. Thus, the increase in myo-inositol levels may result from a composite of suboptimal utilization of myo-inositol, and/or hepatorenal dysfunction.

Elevated plasma levels of glucuronate, erythronate and N6-carbamoylthreonyladenosine levels also were associated significantly with 1-year mortality in patients with PBC or PSC cirrhosis. These results extend our previous findings in patients with cirrhosis caused predominantly by noncholestatic liver diseases [3] to include adults with chronic cholestatic liver diseases. Glucuronate, a carboxylic acid derived from glucose, is required for glucuronidation of substrates in the liver [18–22]. Impaired glucuronidation in patients with advanced cirrhosis [25] results in elevated plasma glucuronate levels [3]. Carbamoylation, a nonenzymatic and irreversible post-translational modification primarily results from the interaction of isocyanic acid with the amino groups of proteins [26–28]. In chronic kidney disease, carbamoylation produces carbamoylated albumin, and carbamoylated fibrinogen [26–28]. Carbamoylation of proteins correlates with renal fibrosis, endothelial dysfunction, increased atherosclerosis, cardiovascular death, oxidative stress and neutrophil dysfunction [26–28]. Thus, elevated plasma levels of N6-carbamoylthreonyladenosine in patients with PBC or PSC cirrhosis who had high liver and kidney disease severity most likely reflect the severity of hepatorenal dysfunction, which in turn significantly associated with increased mortality.

Our results also indicate that plasma metabolite biomarkers can be combined with the MELD-Na score to more accurately predict 1-year mortality. As shown in Table 3 & Figure 3, a combination of several plasma metabolites with MELD-Na score significantly increased the predictive accuracy of 1-year mortality. The top five combined MELD-Na-metabolite models including MELD-Na-myo-inositol, MELD-Na-N6-acetyllysine, MELD-Na-adenosine, MELD-Na-N-acetylputrescine and MELD-Na-N6-carbamoylthreonyladenosine significantly outperformed mortality predicted by MELD-Na score alone, indicating that plasma levels of these metabolites are independently associated with mortality in patients with cirrhosis caused by PBC or PSC. Single-use as well as the combined use of myo-inositol, N-acetylputrescine, trans-aconitate, erythronate, N6-carbamoylthreonyladenosine, N2,N2-dimethylguanosine, glucuronate and dimethylarginine (symmetric dimethylarginine + asymmetric dimethylarginine) with MELD-Na score predicted 1-year mortality more accurately than the MELD-Na score alone (Tables 2 & 3). Additionally, even after controlling for MELD-Na score, several plasma metabolites including myo-inositol, N-acetylalanine, N-acetylputrescine, N6-carbamoylthreonyladenosine, erythronate, N2,N2-dimethylguanosine, glucuronate, creatinine and 4-acetamidobutanoate were significantly associated with 1-year mortality (Table 3).

The physiology of the plasma metabolites including N6-acetyllysine, adenosine, and N-acetylputrescine also are related to the histologic stage of cirrhosis in patients with PBC or PSC (Table 3 & Figure 3). N6-acetyllysine (N-epsilon-Acetyl-L-lysine), that is formed by acetylation of lysine amino acid [18–22], was isolated in rat liver following thioacetamide administration that is a cirrhotic and necrotic agent [29]. In regards to adenosine, it is produced and released in response to injury and hypoxia [30]. Adenosine signals via adenosine A_2A_ receptors on hepatic stellate cells to induce hepatic fibrogenesis [30]. Conversely, adenosine A_2A_ receptor-deficient mice fail to develop hepatic fibrosis in response to carbon tetrachloride and thioacetamide hepatotoxicity [30]. Similarly, selective adenosine A_2A_ receptor antagonists also inhibit fibrogenesis [30]. Altogether, these findings suggest that elevated plasma N6-acetyllysine and adenosine levels in patients with PBC or PSC cirrhosis who had high liver and kidney disease severity reflect severity of fibrosis, portal hypertension, and therefore increased mortality.

N-acetylputrescine is a polyamine formed by the N-acetylation of putrescine by the enzyme diamine N-acetyltransferase [18–22]. Putrescine is synthesized from ornithine by ornithine decarboxylase, the rate-limiting enzyme [18–22,31]. Our results suggest that elevated N-acetylputrescine levels in patients with high liver and kidney disease severity might be due to the upregulation of ornithine decarboxylase mRNA expression in advanced cirrhosis caused by PBC or PSC. Of note, N-acetylputrescine is reported to be one of the unique metabolites of PSC [32].

This study has several methodological strengths. First, it confirmed and extended the utility of the metabolomic signature characterized in our prior analysis of 103 patients with cirrhosis caused mainly by noncholestatic liver diseases [3] in an ‘independent external cohort of patients with cirrhosis caused by PBC or PSC’. Second, it used a time-dependent AUROC analysis instead of using a standard AUROC analysis [15] to evaluate the predictive accuracy of metabolomic biomarkers for mortality. This approach obviated problems caused by standard AUROC analysis in which survival outcomes and metabolite values are considered to be fixed throughout the study period [15]. Third, our application of metabolomics to increase the accuracy of the MELD-Na score is novel. Consideration of precision metabolomic markers is expected to result in a more equitable allocation of donor livers for timely transplantation of patients with cirrhosis caused by PBC or PSC. Our innovative approach lays the groundwork for the incorporation of disease and etiology-specific metabolomic profiles to the MELD-Na score of all other patients with cirrhosis who are at high risk of dying on the waitlist due to their low MELD-Na scores. Fourth, to determine the effect of hepatocellular carcinoma and cholangiocarcinoma on plasma metabolome, we performed a sensitivity analysis by removing the ten patients with hepatic malignancy. Our sensitivity analysis showed that the performance of several single metabolites and bivariate MELD-Na-metabolite models to predict 1-year mortality remained higher than the MELD-Na score alone.

The current study also has inherent limitations. First, only 1-year mortality could be used because too few patients died within 90 days to analyze the predictive value of plasma metabolites alone or in combination with the MELD-Na score on 90-day mortality. Second, the size of the cohort with either PBC or PSC was small to perform gender-specific analyses.

In conclusion, this study shows that plasma concentrations of specific metabolites are accurate biomarkers predictive of 1-year mortality in patients with cirrhosis caused by PBC or PSC. Indeed, the individual plasma metabolites as well as the models combining individual plasma metabolites with the MELD-Na score had greater accuracy in predicting 1-year mortality than the MELD-Na score alone. Plasma metabolite levels alone or in combination with the MELD-Na score provides a novel approach to refine the allocation of donor livers for transplantation to recipients with the highest risk of death on the transplant waiting list. This is particularly germane to PBC, which causes cirrhosis predominantly in women [33]. Women with cirrhosis were shown to have a significantly higher mortality on the liver transplant waiting list compared to men, and this was in part due to the use of serum Cr[34] which is not a gender neutral laboratory variable [35] used in the MELD score to allocate livers.

## Future perspective

Metabolite or MELD-Na-metabolite models can be readily adopted in clinical laboratory medicine. Our innovative approach lays the foundation for the inclusion of disease-specific metabolomic profiles in the MELD-Na system to eliminate inaccuracies of the MELD-Na score. The results of our study should stimulate multicenter prospective validation studies of the reported signature plasma metabolite and combined MELD-Na-metabolite models in larger number of patients with cirrhosis caused by viral, nonviral and autoimmune etiologies, stratified on the basis of sex, men and women. It might also be beneficial to compare the performance of plasma metabolite and combined MELD-Na-metabolite models to UK-PBC [36] and GLOBE [37] risk scores in a future study of patients with PBC cirrhosis. Additionally, a better understanding of specific alterations in metabolism caused by progressive hepatorenal dysfunction may provide insights for new treatment strategies to prevent or modify these metabolic abnormalities in patients with PBC or PSC.

**Summary points**

- We previously identified signature plasma metabolomic biomarkers that were associated with mortality in adults with cirrhosis caused predominantly by noncholestatic liver diseases [3].
- As the cohort in which we identified this metabolomic signature had few patients with cirrhosis caused by cholestatic liver diseases, we tested this signature in an external cohort of patients with two most common adult cholestatic liver diseases, primary biliary cholangitis (PBC) and primary sclerosing cholangitis (PSC).
- Here, we confirm that the specific plasma metabolites of this unique signature are also biomarkers of mortality in adults with PBC or PSC cirrhosis.
- The specific plasma metabolites of this signature were more accurate predictors of 1-year mortality than the model for end-stage liver disease-Na (MELD-Na) score in patients with cirrhosis caused by PBC or PSC.
- Several bivariate combinations of plasma metabolites and MELD-Na score increased the accuracy of predicted 1-year mortality substantially (area under the receiver operating characteristic curve increased from 0.7591 up to 0.9392) in patients with cirrhosis caused by PBC or PSC.
- The significantly higher performance of combined MELD-Na-metabolite models compared with MELD-Na score alone signifies that key metabolite levels are disease-specific biomarkers of prognosis that can amplify prognostic accuracy.
- Plasma metabolites can be used alone or in combination with MELD-Na scores, to accurately predict mortality, minimize underestimations of mortality in patients with cirrhosis and low MELD scores, reduce the risk of death on liver transplant waiting lists and promote equitable allocation of donor livers.
- Plasma metabolomic signatures provide the basis for prospective studies of their predictive value for short-term mortality, alone or in combination with MELD-Na scores, to determine more accurately the risk of death of patients with cirrhosis caused by any etiology.
